# Parental Reactivity to Disruptive Behavior in Toddlerhood: An Experimental Study

**DOI:** 10.1007/s10802-018-0489-4

**Published:** 2018-10-29

**Authors:** Susanne Schulz, Patty Leijten, Daniel S. Shaw, Geertjan Overbeek

**Affiliations:** 1 Child Development and Education and Research Priority Area YIELD, University of Amsterdam, PO Box 15780, 1001 NG Amsterdam, the Netherlands; 2 Research Centre Adolescent Development, Utrecht University, PO Box 80140, 3508 TC Utrecht, the Netherlands; 3 University of Pittsburgh, 4101 Sennott Square, 210 South Bouquet Street, Pittsburgh, PA 15260 USA

**Keywords:** Disruptive behavior, Self-efficacy, Stress, Arousal, Individual differences, Positive parenting

## Abstract

Disruptive child behavior is often exacerbated and maintained by negative and inconsistent parenting behavior that unwittingly reinforces disruptive behavior. One explanation for why parents render it difficult to remain positive and consistent might be the impact of disruptive child behavior on parent self-efficacy and stress. This study investigates how disruptive child behavior in a challenging parenting situation shapes parental momentary thoughts of self-efficacy and feelings of stress (i.e., perceived distress and physiological arousal), and how these in turn predict parenting behavior. We experimentally manipulated a challenging parenting situation that was designed to elicit disruptive child behavior. Specifically, we examined: (1) the effects of the challenging condition compared to a control situation on parental state self-efficacy and stress, (2) whether parents with lower trait self-efficacy and higher trait stress in daily life are most affected, and (3) how state self-efficacy and stress predict parental subsequent use of direct commands and positive affect. Parent-toddler dyads were randomly assigned to a challenging or control situation (*N* = 110, *M*_age_ = 30.9 months). As predicted, parents in the challenging situation, relative to control, reported less self-efficacy and more perceived distress, and showed increased physiological arousal. Self-efficacy was compromised particularly in parents with low trait self-efficacy. Our findings suggest that child disruptive behavior drives parental state self-efficacy and stress, especially momentary self-efficacy in parents who generally feel less self-efficacious.

**Electronic supplementary material:**

The online version of this article (10.1007/s10802-018-0489-4) contains supplementary material, which is available to authorized users.

The transition from infancy to toddlerhood marks a period in which children become increasingly disruptive. They show more oppositional behavior and are less likely to comply with a parental request (Shaw and Bell 1993). Such behavior is typically a sign of healthy development, and reflects the development of autonomy in the child (Scaramella and Leve 2004). When disruptive child behavior persists, however, it puts children at risk for mental health problems in later life (Tremblay 2000). Persistent disruptive behavior is often maintained and exacerbated by negative and inconsistent parenting, which inadvertently reinforces disruptive child behavior (Patterson 1982). In this study, we explored the processes underlying parental reactions to disruptive child behavior. We proposed that parental thoughts of self-efficacy and feelings of stress evoked by disruptive child behavior may explain why parents adopt more negative and inconsistent parenting behavior. Specifically, we investigated situational self-efficacy and stress in response to disruptive child behavior, and how these reactions in turn would predict parenting behavior. Understanding the processes underlying parental reactions to disruptive child behavior could refine our understanding about the development of parent-child interactions, and might help to optimize parenting interventions that help parents to better manage disruptive child behavior.

## Effects of Disruptive Child Behavior on Parental “State” Self-Efficacy and Stress

States represent thoughts and feelings at a specific point in time that are variable, temporary, and evoked by an external situation (Chaplin et al. 1988). Traits, on the other hand, are relatively stable over a period of time, enduring, and often evoked internally. While trait and state levels are usually associated, in that traits are often inferred from cumulated state experiences across different situations over time, they are also meaningfully different (Spielberger 1972). That is, while parents’ general levels of self-efficacy and stress (i.e., traits) are relatively persistent over a period of time, their immediate levels of self-efficacy and stress (i.e., states) fluctuate and differ depending on the experienced situation or stressor. For example, parents might generally experience high levels of self-efficacy and low levels of stress in their daily life, but still feel less self-efficacious and highly stressed in reaction to a specific challenging situation. In this state of low self-efficacy and high stress, parents might behave differently than they usually would.

### Parental “State” Self-Efficacy

One reason why parents might experience disruptive child behavior as particularly challenging is that such behavior affects their self-efficacy. Thoughts of self-efficacy reflect how competent parents perceive themselves in their parenting role and their abilities to manage parenting situations (Bandura 1977; Teti and Gelfand 1991). Especially in challenging situations that elicit disruptive child behaviors, parents may experience increased difficulty and unsuccessful attempts to manage the child’s behavior, which could evoke momentary thoughts of incompetence (Lipscomb et al. 2011; Porter and Hsu 2003). Parents who experience such low self-efficacy, in turn, more easily get frustrated and persist less in challenging situations, which can negatively impact their parenting practices in toddlerhood (Coleman and Karraker 1997). For example, previous findings show that less self-efficacious parents are less likely to engage in positive parenting, such as positive affect, and more likely to engage in negative parenting, such as harsh punishment (Jones and Prinz 2005; Mouton and Roskam 2015). Thus, it is important to understand how parental self-efficacy may be altered and shaped by specific parenting situations in toddlerhood. However, studies on parental self-efficacy typically do not investigate parental state self-efficacy in response to an actual parenting situation, but rather assess parental trait self-efficacy, which can mask the actual effects of disruptive child behavior on parental state self-efficacy. As parental trait self-efficacy might be quite resistant to transient events, it is recommended to examine situation-dependent fluctuations in parental self-efficacy, using intensity scales and items that capture momentary feelings (Spielberger 1972). Furthermore, most research relies on correlational designs that do not allow to investigate the immediate causal effects of disruptive child behavior on parental state self-efficacy. This study implemented an experimental design to test the immediate, causal effects of a challenging parenting situation, which was designed to elicit disruptive child behavior, on parental state self-efficacy.

### Parental “State” Stress

Another reason why parents might find it difficult to manage their child’s disruptive behavior concerns their immediate stress reactions to disruptive child behavior. Stress is an emotional reaction to an event that is perceived as threatening (Lazarus and Folkman 1984). This stress reaction constitutes different components, such as subjective experiences that refer to the perceived feelings of distress, and arousal that refers to the physiological activation and intensity of a stress response (Lazarus and Folkman 1984; Scherer 2005). Particularly situations in which children become disruptive are often perceived as irritating and stressful (Crnic and Low 2002). These aversive situations require parents’ immediate attention, which can overwhelm and distress them (Goldstein et al. 2007). When parents experience such high levels of acute stress and arousal, in turn, they more easily get irritated and less focused on the child’s needs, which compromises their ability to engage in positive parenting (Dix 1991; Eisenberg and Fabes 1994; Leerkes et al. 2016).

Subjective and physiological components of a stress response can function independently (Laurent et al. 2013; Scherer 2005). Hence, parents may show physiological arousal without perceiving a situation as particularly stressful. Nonetheless, this arousal can affect their ability to regulate their behavior and consequently, their use of positive parenting practices. Self-reports provide insight into perceived feelings, but they do not permit investigation of physiological arousal. The current study, therefore, investigated two components of parental stress reactivity: subjective feelings that indicate how parents perceive a challenging parenting situation, such as irritating or tense, and arousal that describes how parents respond physiologically to a challenging situation.

Arousal can be non-invasively observed with changes in skin conductance levels (SCL) that are widely used, valid indicators of emotional states in stress-inducing situations (Boucsein 1992; Brouwer and Hogervorst 2014; Kreibig 2010). Measures of SCL assess increased activity in the sweat glands, which reflect activation of the sympathetic nervous system to promote a behavioral response (Boucsein 1992). Parents can experience excessive arousal, as indexed by increased SCL, in response to various external stressors, such as recorded infant crying (Frodi et al. 1981; Groh and Roisman 2009). Such excessive SCL may impede parents’ functioning and has been repeatedly linked to harsh, abusive parenting (Frodi and Lamb 1980; Joosen et al. 2013). However, previous studies investigated arousal in response to child behavior detached from actual challenging situations. The present study expands previous research by investigating parental perceived distress and physiological arousal in challenging parenting situations.

## Individual Differences in Parental Reactivity to Disruptive Child Behavior

Not all parents are affected equally by disruptive child behavior. While some parents may become less self-efficacious and more stressed when children show disruptive behavior, other parents remain relatively calm and positive (e.g., Leerkes et al. 2016). One possible explanation for these individual differences might be that parents who in daily life experience lower levels of trait self-efficacy and higher levels of trait stress (i.e., enduring feelings of perceived distress and elevated base levels of physiological arousal) are more vulnerable to the effects of disruptive child behavior. Persistently doubting their own abilities and exceeding their own resources impedes parents’ daily functioning and their reactions to an immediate stressor (Lazarus and Folkman 1984; Spielberger 1972). As such, lower levels of trait self-efficacy and higher levels of trait stress might lead parents to respond with overly strong reactions to disruptive child behavior (Ardelt and Eccles 2001; Joosen et al. 2013). Stronger reactions in turn might make it particularly difficult for parents to recover from challenging situations and thus affect their parenting behaviors (Joosen et al. 2013; Sturge-Apple et al. 2011). The current study therefore investigated whether parental levels of trait self-efficacy and trait stress increase the effects of disruptive child behavior on parental state levels of self-efficacy and stress, respectively.

## Effects of Parental Reactivity to Disruptive Child Behavior on Parenting

Ample findings indicate that disruptive child behaviors negatively impact parenting behaviors that are relevant to the maintenance of positive parent-child interactions (Gardner et al. 1999; Scaramella and Leve 2004; Verhoeven et al. 2010). If parents fail to use positive parenting strategies, they may inadvertently reinforce disruptive child behavior and pave the way for coercive interactions (Patterson 1982; Shaw et al. 1994; Smith et al. 2014). However, much remains unknown about the mechanisms through which disruptive child behavior decreases parents’ use of positive parenting strategies. As parental self-efficacy and stress are related to positive parenting skills, they may mediate the effects of disruptive child behavior on positive parenting. Initial studies suggest that higher levels of child behavior problems predict less positive parenting practices through decreased levels of parental self-efficacy (Day et al. 1994) and increased levels of parental perceived distress (Goldstein et al. 2007) as well as arousal (Joosen et al. 2013).

Most studies on positive parenting used broad categories that comprise multiple behaviors, such as affection, praise, rewards, and support (e.g., Gardner et al. 2007; Miller et al. 2015). Although these categories are helpful to distinguish between more positive and more negative parenting behaviors, the use of broad categories cannot identify specific parenting strategies that are most (or least) affected by disruptive child behavior. The present study therefore examined how parental state self-efficacy and stress in challenging situations translate to two positive parenting strategies: direct commands and positive affect. Direct commands are anticipatory, rather than merely reactive, applied strategies that allow parents to prevent and manage disruptive child behavior by communicating clear expectations (Eyberg and Robinson 1982; Gardner et al. 2007). Positive affect is a continuous strategy with which parents create a supportive environment by helping children to elicit positive emotions and behaviors (Dix 1991; Eisenberg et al. 2005). We focused on these strategies as verbal and non-verbal dimensions of positive parenting because direct commands help parents to control and direct their child’s behavior, while positive affect helps parents to direct their child’s emotions and prevent disruptiveness. Together, they can facilitate cooperative behaviors in children.

## The Present Study

**Fig. 1:**
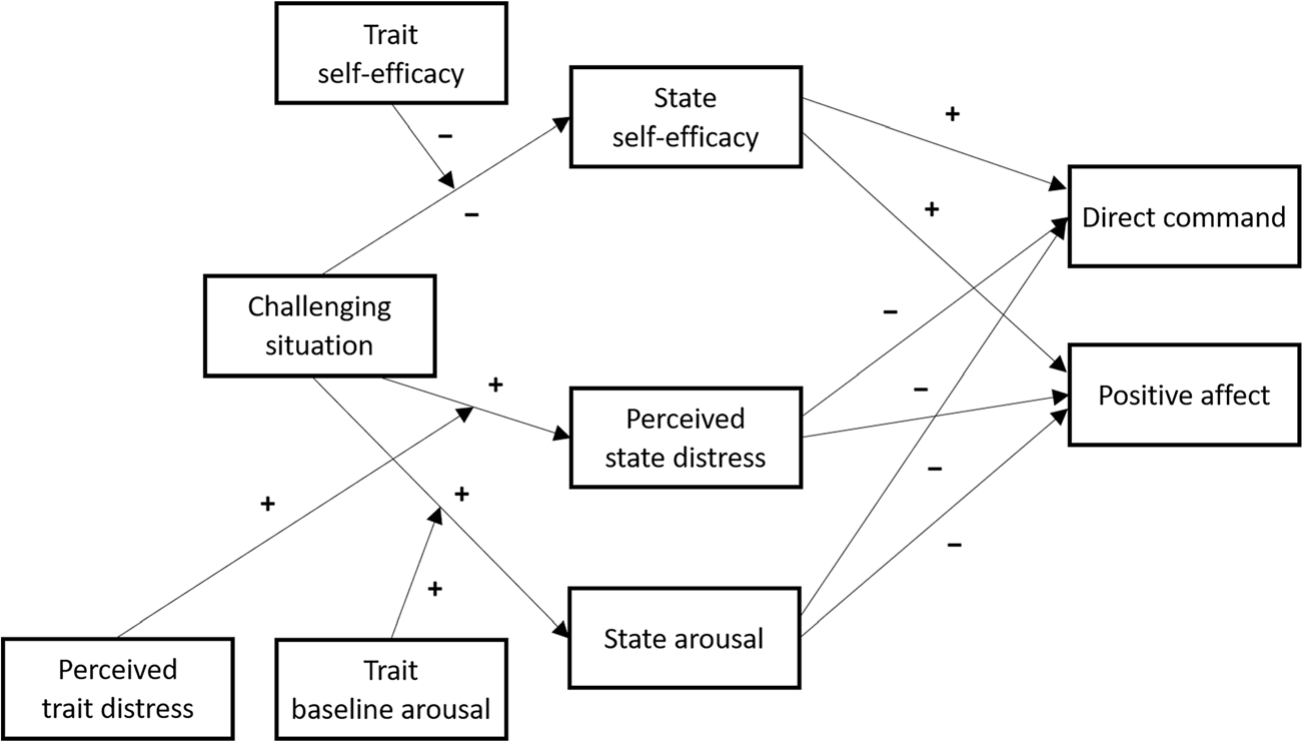
Conceptual model

We examined parental state self-efficacy and state stress, indicated by perceived distress and arousal, in response to a challenging parenting situation―characterized by disruptive child behavior―in an experimental design. In addition, we tested whether parental trait self-efficacy and trait stress, indicated by perceived trait distress and baseline arousal, moderated parental state self-efficacy and stress in a challenging situation. Lastly, we tested how levels of parental state self-efficacy and stress in response to disruptive child behavior translated to parents’ use of direct commands and positive affect. Our hypotheses were threefold: (1) Parents were expected to show lower levels of self-efficacy and higher levels of perceived distress and arousal when they experienced a challenging situation compared to a non-challenging control situation; (2) Lower levels of trait self-efficacy, higher levels of perceived trait distress, and higher levels of baseline arousal were expected to increase the effects of disruptive child behavior on parental state self-efficacy, perceived distress, and arousal, respectively; and (3) The challenging situation was expected to indirectly reduce parental use of direct commands and positive affect by decreasing levels of parental state self-efficacy and increasing levels of parental perceived distress and arousal (see Fig. 1).

## Methods

### Participants

The sample consisted of 110 parents and their two-year-old toddlers (*M*_age_ = 30.89 months; *range* = 24 to 36 months, 46.4% female). The majority of parents were mothers (84.5%), were living together with a partner (93.6%), and had a high educational background (63.6% university, 29.0% vocational). Parents were on average 36.2 years of age, born in the Netherlands (80%) and spoke Dutch (80.9%) or English (7.3%) to the child. Only few parents had received professional help for the child’s behavioral problems (5.5%). As we specifically targeted families with disruptive children, toddlers showed significantly higher levels of disruptive behavior than average Dutch children, *t*(171.09) = 8.39, *p* < 0.001 (Weeland et al. 2017). Families in the challenging and control condition did not differ significantly on any demographic characteristics (*p*s > 0.194).

### Procedure

Parents were recruited through a database of the University of Amsterdam. This database contains families who responded to letters that invited parents with newborns in the city to participate in studies by the university. Approximately one to three months before the laboratory visit, parents with a child in the age group within 21 and 33 months of age (*N* = 425) were asked to report on the frequency of their child’s disruptive behavior problems, such as noncompliance and aggressiveness, using an online version of the Eyberg Child Behavior Inventory (ECBI; Eyberg and Pincus 1999). More than half of these families (*n* = 231, 54.35%) completed the questionnaire. Based on a priori power analyses to detect medium to large effects with a probability of .80, we selected the 138 families with the highest scores on disruptive child behavior (score > 90 on the ECBI Intensity scale, resulting in a sample mean that roughly corresponds to the 75th percentile of disruptive behavior levels in Dutch children). This approach ensured sufficient and varying levels of disruptive behavior during the study to examine how parents respond to this behavior. Twenty-eight families dropped out because they did not respond to our attempts to schedule a lab visit (*n* = 13), or because they cancelled their appointments and were not able to reschedule before the child reached 36 months in age (*n* = 15). Dropped out families reported slightly higher levels of disruptive child behaviors than participating families, but this difference did not attain statistical significance, *t*(136) = 1.84, *p* = 0.068.

Parents provided written informed consent for themselves and their children. About one week before the laboratory visit, parents completed an online survey that assessed their levels of trait self-efficacy and stress. If necessary, parents received reminders via email or telephone to ensure that all participants completed the survey before their laboratory visit. Families were randomly assigned to a challenging (*n* = 56) or control condition (*n* = 54). They were unaware of the specific aims of this research and were told that the study investigated parent-child interactions in different play situations. After parents had been informed about the general procedures, an experimenter assessed parents’ baseline arousal. All subsequent parent-child interactions were video-recorded and observed through a one-way mirror.

**Fig. 2:**
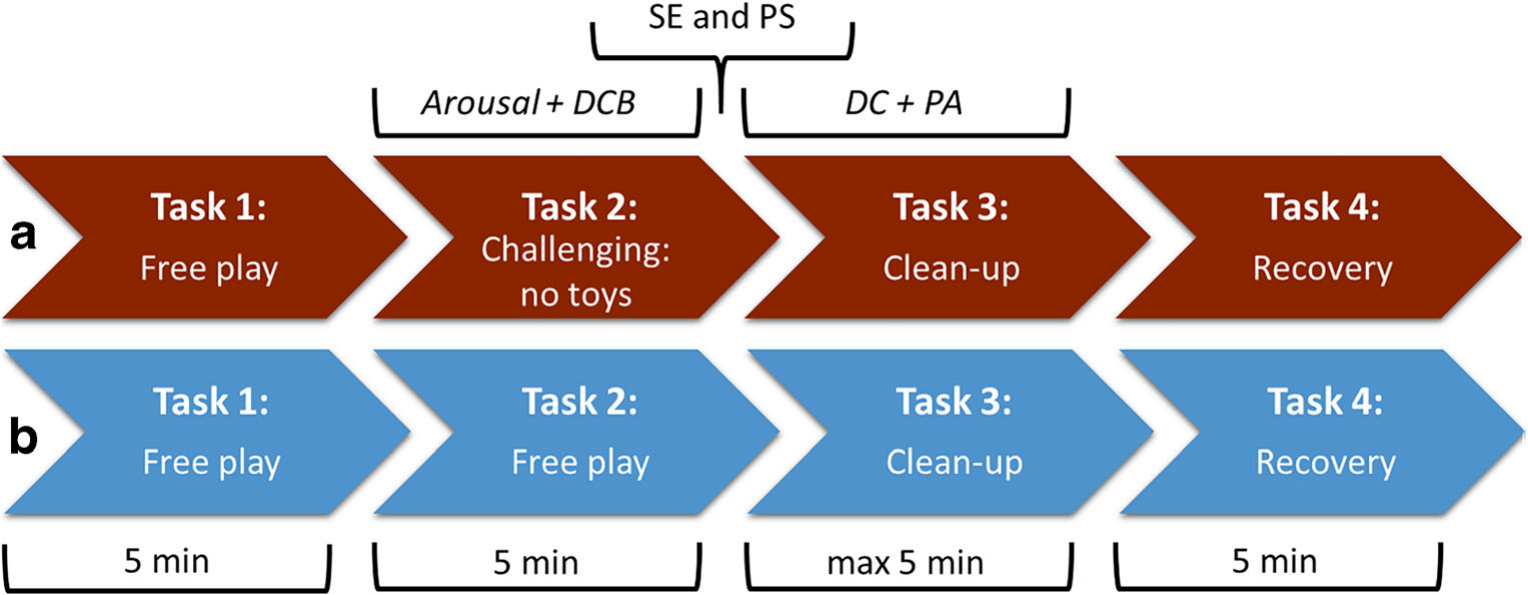
Sequence of the challenging (**a**) and control condition (**b**), including durations and parents’ assessments during the tasks: DCB = disruptive child behavior, SE = self-efficacy, PS = perceived distress, DC = direct commands, PA = positive affect

The experiment consisted of four tasks (see Fig. 2). These tasks have been previously used to examine challenging and non-challenging parenting situations in toddlerhood (Martin 1981; Shaw et al. 1994). First, parents and children engaged in a free play situation with toys to make them feel comfortable and provide equal starting conditions. Second, in the challenging condition, the parent was instructed to clean up all toys into a transparent box and complete a questionnaire consisting of more questions than the parent could manage to complete. In the control condition, the parent continued to play with the child and the toys. Thus, the challenging condition represented a challenging parenting situation, which was designed to elicit disruptive child behavior because of a double challenge: (1) Children were prohibited from playing with the (visible) toys; and (2) children were denied their parents’ attention who needed to perform a different task. Third, the experimenter re-entered the room with a new set of motivating toys. The experimenter engaged the child in playing with these toys, while the parent completed a short questionnaire regarding their feelings of self-efficacy and stress during the previous task. The end of this questionnaire provided the instructions for the following task, in which the parent requested the child to clean up the new set of toys without providing any assistance. This task was designed to observe how parents’ responses to challenging situations would translate to their use of direct commands and positive affect. Fourth, to end the experiment positively, parent and child engaged in a pleasant free-play activity. After the experiment, parents were debriefed about the specific purposes of this study, including the challenging parenting situation that they could have perceived as stressful. The study procedure was approved by the Ethics Review Board of the University of Amsterdam.

### Measures

#### Parental State Self-Efficacy

We assessed state self-efficacy as parents’ self-reported confidence and performance during the (non-)challenging situation (see Online Resource 1). The brief project-developed questionnaire consists of six statements (e.g., “I managed the task well”) that were adapted from previously validated questionnaires to assess parents’ state self-efficacy in specific situations (Johnston and Mash 1989; Moran et al. 2016). Parents rated each statement on a 5-point intensity scale (1 = not at all; 5 = to a large extent). The scale showed good internal consistency (α = 0.87).

#### Parental State Stress

We assessed parental state stress through (1) self-reported perceived distress and (2) physiological arousal during the (non-)challenging situation. Parents reported on their levels of perceived distress, using a brief project-developed questionnaire (see Online Resource 1). The questionnaire consists of seven statements (e.g., “I felt stressed”) that were adapted from previously validated questionnaires to assess feelings of state stress in a particular situation (Duckro et al. 1989; Levenstein et al. 1993). Parents rated each statement on a 5-point intensity scale (1 = not at all; 5 = to a large extent). The scale showed good internal consistency (α = 0.88).

We assessed parental arousal through changes in parental skin conductance levels (SCL). Electrodermal activity was measured using two curved 20 × 16 mm Ag/AgCl electrodes attached to the medial phalanges of the parents’ index and middle finger of their non-writing hand. SCL were recorded at a sampling rate of 50 Hz with Versatile Stimulus Response Registration Program (Vsrrp98 v7.0; Technical Support Group of the Department of Psychology, University of Amsterdam). SCL scores during the (non-)challenging situation were averaged and divided by individual baseline scores to calculate difference scores in arousal. Higher values indicate increased levels of arousal from baseline to the (non-)challenging situation. This measure has been frequently used to measure stress responses in parents (Joosen et al. 2013; Leerkes et al. 2016).

#### Parental Trait Self-Efficacy

We assessed trait self-efficacy with parents’ self-reports on the efficacy subscale of the Parenting Sense of Competence (PSOC; Johnston and Mash 1989). It consists of seven items, which address the extent to which parents feel competent in their parenting role (e.g., “If anyone can find the answer to what is troubling my child, I am the one”). Parents rated their thoughts in the past three weeks on a 6-point scale (1 = strongly disagree; 6 = strongly agree). The scale showed adequate internal consistency in the present sample (α = 0.76).

#### Parental Trait Stress

We assessed trait stress through (1) self-reported persistent stress in daily life and (2) baseline arousal. Parents reported on their perceived levels of trait distress, using the stress subscale of the Depression Anxiety Stress Scale (DASS; Lovibond and Lovibond 1995). It consists of ten items, which address persistent irritability and nervous tension in the last three weeks (e.g., “I found it difficult to relax”). Parents rated their feelings on a 4-point scale (1 = did not apply to me at all; 4 = applied to me very much, or most of the time). The scale demonstrated high internal consistency in the present sample (α = 0.91).

We assessed baseline arousal through parental skin conductance levels during their resting states for two to three minutes. Higher values indicate higher levels of baseline arousal. SCL resting states have been previously used to assess chronic arousal levels (Reijman et al. 2016).

#### Direct Commands

We observed parental use of direct commands by coding how parents instructed their children to clean up in the third task. Direct commands (e.g., “Please put away the toys”) clearly communicate parental expectations and thus, make it easier for children to comply, whereas indirect commands such as questions (e.g., “Do you want to put away the toys?”) or vague suggestions (e.g., “Make the room nice and tidy”) do not clearly request compliance and thus, make it more difficult for the child to understand parental expectations. Coding was based on the frequently used Dyadic Parent–Child Interaction Coding System (DPICS; Eyberg and Robinson 1981): A command was coded as direct if it was stated positive (i.e., telling the child what to do, instead of telling the child what not to do) and direct (i.e., clear and specific). Three raters who were blind to conditions analyzed parents’ initial instruction as either direct (1) or not (0). Interrater agreement based on 30% of the sample showed good agreement (κ = 0.87).

#### Positive Affect

Parental positive affect was observed during the clean-up task. Based on the DPICS coding manual, proportions of positive affect were analyzed as the total number of present expressions of enjoyment, enthusiasm, and warmth directed at the child in five second intervals divided by the total number of intervals. Higher values indicate higher levels of positive affect. Three raters who were blind to conditions assessed positive affect. Interrater agreements using interclass correlations (ICC) based on 30% of the sample showed acceptable agreement (ICC = 0.80).

#### Disruptive Child Behavior during the (Non-)Challenging Task

As a manipulation check, we coded the number of child disruptive incidents during the (non-)challenging situation to verify that children in the experimental condition indeed showed more disruptive behavior than children in the control condition. Disruptive behaviors were coded based on previous studies (e.g., Shaw et al. 1994) and included nagging, crying, attempting to leave, being destructive or aggressive, or not following parents’ commands. Two raters who were blind to conditions coded disruptive child behavior as present or absent in ten second intervals. The total score refers to the total number of intervals including disruptive incidents. Interrater agreements based on 10% of the sample showed perfect agreement (ICC = 0.99).

### Analytic Strategy

Analyses consisted of three steps in line with our hypotheses. First, we conducted three ANOVAs to test the effects of the challenging situation on parental state self-efficacy, perceived state distress, and arousal. Second, we conducted regression analyses to test whether parental trait self-efficacy, perceived trait distress, and baseline arousal interact with the manipulation, such that these traits increase the effects of the challenging situation on parental state self-efficacy, perceived state distress, and arousal, respectively. Third, we used Structural Equation Modeling (SEM) to test whether state self-efficacy, perceived state distress, and arousal mediate the effects of a challenging parenting situation on parental use of direct commands and positive affect. The package Lavaan (Rosseel 2012) for the software program R 3.5.0 (R Core Team 2018) was used to fit the proposed path model to the data. To estimate model parameters and evaluate model fit, we used diagonally weighted least squares with robust variants. We handled missing values with listwise deletion, because our categorical outcome did not allow full information maximum likelihood estimation, and determined model fit using several fit indices: the chi-square test of model fit, the comparative fit index (CFI), and the root mean squared error of approximation (RMSEA). A significant chi-square value indicates a significant discrepancy between the model-implied and the observed covariance matrix. CFI values > 0.95 indicate good fit. RMSEA values < 0.05 indicate close fit, values < 0.08 indicate adequate fit, and values > 0.10 indicate poor fit (Hu and Bentler 1999). Where model modification was necessary, we considered correlation residuals > 0.10 substantial.

## Results

### Preliminary Analyses

Missing data was very low (0.43% across all variables). Little’s missing completely at random (MCAR) test detected no systematic patterns of missingness, χ^2^ (2737) = 190.734, *p* > 0.999, which indicates that missing data was not likely to produce any bias in the analyses. Some data were omitted from the analyses because of violations to the study procedures: for state self-efficacy and stress because another child was present during the (non-)challenging situation (*n* = 1), for arousal because of equipment failures (*n* = 3), for trait self-efficacy and stress because parents did not complete the questionnaire (*n* = 3), for parental use of direct commands because parents failed to give a command or because children started to clean up prior to the parents’ command (*n* = 4). Mild outliers (> 1.5 *SD*) were detected for all variables, except baseline arousal, and were replaced by the highest non-outlying value of the distribution. We detected no further major violations against the assumptions of the analyses.

Table 1 displays descriptive statistics and correlations among all variables. We calculated Pearson correlations for continuous variables, polyserial correlations for continuous and dichotomous variables, and polychoric correlations for dichotomous variables. State self-efficacy and state distress correlated strongly and significantly, indicating that while they may reflect different constructs, they are also interrelated. Parental use of direct commands and positive affect also correlated significantly, indicating that parents who used more direct commands also showed more positive affect. This was expected as they represent two distinct positive parenting strategies.

**Table 1:** Correlations between all study variables for both groups

Variable	*M*	*SD*	1	2	3	4	5	6	7	8
1 Challenging situation										
2 State self-efficacy	4.30	0.64	−0.47***							
3 State distress	1.73	0.59	0.49***	−0.52***						
4 Arousal	1.57	0.31	0.36**	−0.09	0.12					
5 Trait self-efficacy	4.30	0.70	−0.05	0.37***	−0.16	0.10				
6 Trait distress	1.54	0.41	−0.03	−0.18	0.26**	−0.25*	−0.33***			
7 Baseline arousal	9.04	3.94	0.01	−0.05	0.14	−0.16	−0.03	0.17		
8 Direct command	0.20	0.40	0.39*	−0.04	0.06	−0.05	−0.05	0.14	−0.02	
9 Positive affect	0.23	0.17	0.15	0.03	0.02	0.04	0.05	0.19	0.13	0.34**

*** *p* < 0.001 ** *p* < 0.01 * *p* < 0.05

Split correlations for the experimental and control group separately can be found in Online Resource 2

The manipulation successfully led to more disruptive child behavior in the challenging condition than in the control condition (*t*(60.995) = −6.75, *p* < 0.001, Cohen’s *d* = 1.27). Specifically, children in the challenging condition displayed on average approximately fourteen times as many instances of disruptive behavior as children in the control condition.

### Main Analyses

#### Hypothesis 1: Disruptive Child Behavior during the Challenging Situation Leads to Lower Parental State Self-Efficacy and Higher State Stress

**Fig. 3:**
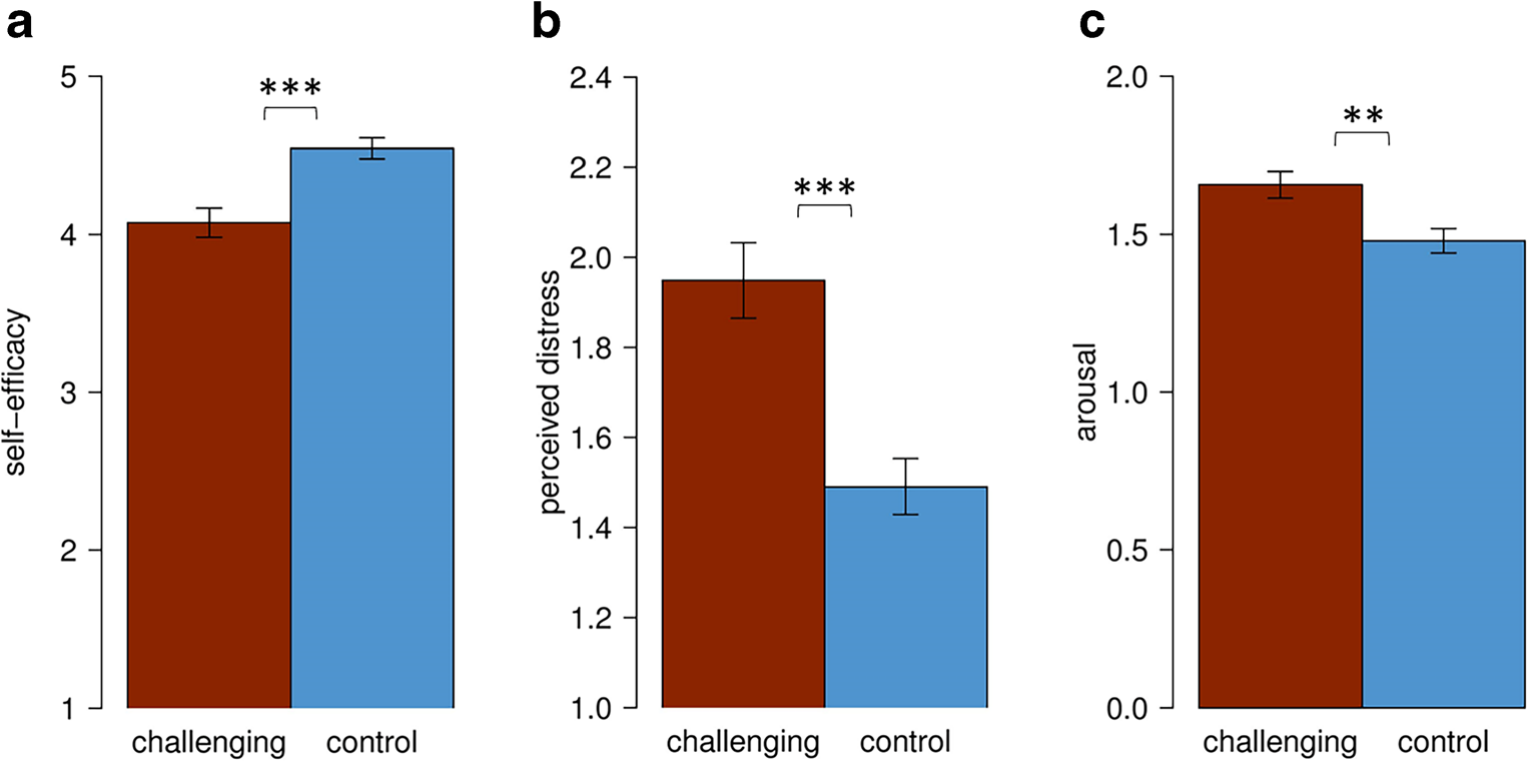
The effects of the challenging vs. control situation on parental state self-efficacy (**a**), perceived state distress (**b**), and arousal (**c**) *** *p* < 0.001 ** *p* < 0.01

As expected, the challenging situation was associated with differences in parental state self-efficacy and state stress. Specifically, ANOVAs (Bonferroni-adjusted α = 0.017) revealed that parents in the challenging condition, compared to the control condition reported significantly lower state self-efficacy (*F*(1, 107) = 16.55, *p* < 0.001, *d* = 0.78) and higher perceived distress (*F*(1, 107) = 19.16, *p* < 0.001, *d* = 0.84), and showed increased arousal (*F*(1, 104) = 9.31, *p* = 0.003, *d* = 0.59; Fig. 3).

#### Hypothesis 2: Parental Trait Self-Efficacy and Trait Stress Moderate the Effects of Disruptive Child Behavior on Parental State Self-Efficacy and State Stress

**Fig. 4:**
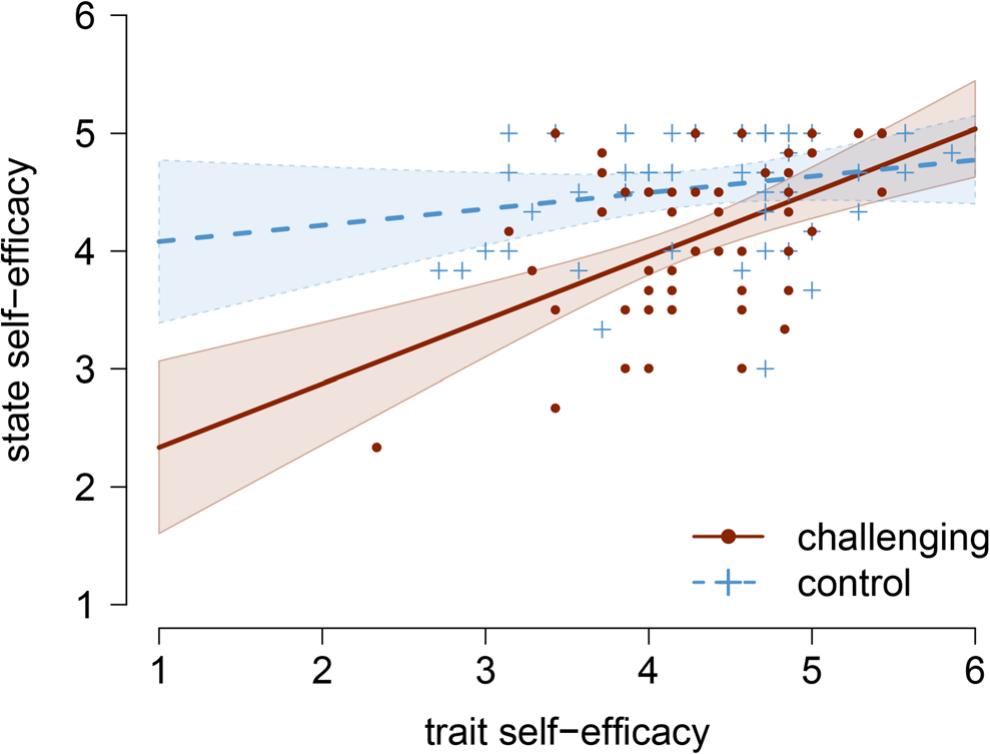
Interaction effects of the challenging situation and parental trait self-efficacy on parental state self-efficacy

Consistent with our expectations, trait self-efficacy significantly moderated the effect of the challenging situation on parental state self-efficacy (*b* = 0.40, *t*(102) = 2.67, *p* = 0.009, η^2^ = 0.065, *R*^2^ = 0.28). However, perceived trait distress and baseline arousal did not moderate the effect of the challenging situation on parental perceived state distress (*b* = −0.41, *t*(102) = −1.67, *p* = 0.098, η^2^ = 0.027) and arousal (*b* = −0.02, *t*(102) = −1.49, *p* = 0.139, η^2^ = 0.021), respectively. The manipulation thus compromised state levels of self-efficacy particularly in parents who had low levels of trait self-efficacy (Fig. 4).

#### Hypothesis 3: Parental State Self-Efficacy and State Stress Mediate the Link between Disruptive Child Behavior and Parenting Behaviors

A table depicting model comparisons can be found in Online Resource 3. The baseline mediation model failed to adequately predict how the challenging situation influenced parents’ use of direct commands and positive affect through their state self-efficacy and stress, χ^2^(6, *N* = 102) = 35.142, *p* < 0.001, RMSEA = 0.219, CFI = 0.500. We therefore successively added a covariance between state self-efficacy and state distress, and between direct commands and positive affect, to account for their high correlation residuals (−0.51 and 0.32, respectively). This modified second model provided approximate fit based on some but not all fit indices, χ^2^(4, *N* = 102) = 8.108, *p* = 0.088, RMSEA = 0.101, CFI = 0.930. Model inspections indicated another high correlation residual (0.70) between the challenging situation and parents’ direct commands. We therefore added a direct effect of the challenging situation on parents’ direct commands. This model was superior to all previous models, providing good fit based on all fit indices, χ^2^(3, *N* = 102) = 1.619, *p* = 0.655, RMSEA < 0.001, CFI > 0.999, *R*^2^ = 0.16, and was accepted as our final model.

**Fig. 5:**
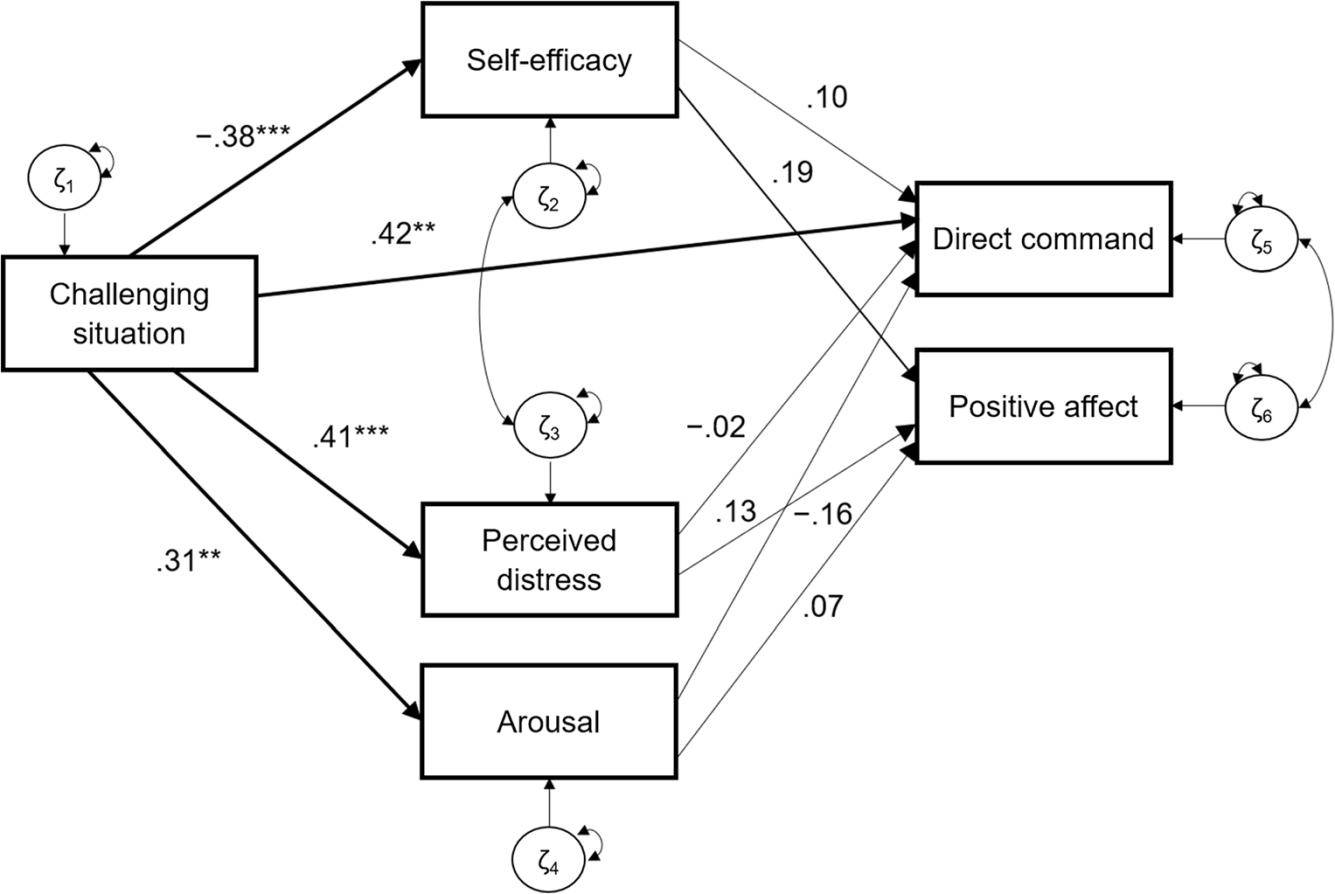
Final model with standardized parameter estimates. χ^2^(3, *N* = 102) = 1.619, *p* = 0.655, RMSEA < 0.001, CFI > 0.999. Squares represent observed variables; one-sided arrows direct effects between variables; double-sided arrows covariances. Circles represent residual factors ζ. * *p* < 0.05. ** *p* < 0.01. *** *p* < 0.001

Figure 5 displays the final model. Confirming the results of the separate ANOVAs, parents in the challenging, compared to the control, situation reported lower self-efficacy and higher stress and showed more arousal. Contrary to our predictions, parental state self-efficacy, perceived distress, or arousal neither predicted decreases in positive affect nor in use of direct commands. Although we detected a trend indicating that parental state self-efficacy increased parental use of positive expressions on average by 18.5%, this effect did not reach significance. Further, all mediation effects were negligible and not statistically significant (*p*s > 0.10). Contrary to our expectations, parents in the challenging condition used significantly more, instead of fewer, direct commands than parents in the control condition.

Sensitivity analyses including non-adjusted outlying values revealed no differences for any of the study outcomes. We further conducted sensitivity analyses that corrected for missing data, by using multiple imputations on missing items. None of our results changed, indicating that missing data did not bias our findings. Finally, including parent gender, child gender, parent age, parent education, parent country of birth, and family constellation as covariates did not change any of the study results. However, adding child age as a covariate led to a significant positive association between parental state self-efficacy and parental positive affect, β = 0.23, *p* = 0.025, including a small mediation effect, β = −0.09, *p* = 0.050, in the final mediation model. This suggests that lower parental state self-efficacy in challenging situations might indeed predict less positive affect, when accounting for child age.

## Discussion

Negative parent-child interactions are a key mechanism underlying the development and maintenance of disruptive child behavior, especially during early childhood (Martin 1981; Shaw et al. 1994). Much remains unknown about why parents use specific parenting strategies to manage disruptive child behavior, such as clear commands and positive affect, as opposed to negative strategies, such as inconsistent commands and negative affect. The present study investigated the effects of a challenging situation that elicits disruptive child behavior on parental immediate thoughts of self-efficacy and feelings of stress, and whether these effects are most pronounced in parents with low trait self-efficacy and high levels of trait stress in their daily life. We found that the challenging situation indeed compromised parental self-efficacy and increased parental stress compared to a non-challenging situation. Parents with low trait self-efficacy were most vulnerable to the adverse effects of challenging situations on thoughts of self-efficacy.

### Parental Reactivity to Disruptive Child Behavior

Consistent with our first hypothesis, our findings extend previous studies that found associations of disruptive child behavior with parental self-efficacy (Lipscomb et al. 2011), perceived distress, and arousal (Frodi et al. 1981; Groh and Roisman 2009). Adopting an experimental paradigm with situationally induced disruptive child behavior, our findings show that a challenging, compared to a non-challenging, parenting situation simultaneously causes parents to feel less self-efficacious, and more stressed and aroused. Thus, challenging situations that elicit disruptive behavior are likely to alter parents’ momentary states of self-efficacy and stress because of experiences of repeated failure and perceived threat (Porter and Hsu 2003; Lazarus and Folkman 1984).

Consistent with our second hypothesis, particularly parents with low levels of trait self-efficacy reported less state self-efficacy during the challenging, compared to the control, situation. As traits often reflect cumulated state experiences in various situations, trait levels are likely to moderate and elevate state reactions (Lazarus and Folkman 1984; Spielberger 1972). Parents with low trait self-efficacy might perceive repeated instances of disruptive child behavior as failures to their parenting attempts, doubting that they possess the means to successfully manage disruptive child behavior. If these parents do not expect their continuous efforts to be successful, they might engage in fewer attempts to effectively respond to their child’s behavior, and thus potentially risk negative parent-child interactions.

While parents with low trait self-efficacy also showed stronger negative self-efficacy reactions to the challenging situation, parents with high trait stress did not show stronger negative stress reactions to the challenging situation. Parental perceived distress and physiological arousal may be more strongly bound to situational circumstances, which relates to the variable and relatively brief nature of immediate stress reactions (Laurent et al. 2013; Scherer 2005). Past distress and chronic arousal might therefore be less likely to impact current parental stress reactions to challenging situations.

Although sensitivity analyses suggested partial support for our third hypothesis, parental state self-efficacy and state stress reactions to disruptive child behavior did not predict parental use of direct commands or positive affect. While theoretical and empirical studies associate state self-efficacy and stress with less positive parenting behaviors (Mouton and Roskam 2015; Coleman and Karraker 1997; Leerkes et al. 2016), our findings suggest that there may be time constraints on the separation of parental state responses and parenting behavior. We investigated parenting strategies in a clean-up task that took place *after* the challenging situation to allow for temporal associations between disruptive child behavior and subsequent parenting strategies. As states are transient and bound to momentary events (Chaplin et al. 1988), the clean-up task may represent a situation that parents newly appraise, relatively irrespective of their experiences in the previous task. Rather than their feelings from the challenging situation persisting onto the new situation, parental situational thoughts and feelings during the clean-up task may influence their use of parenting strategies.

Although neither state self-efficacy nor state stress predicted subsequent parenting practices, parental use of direct commands was directly shaped by the challenging situation. Contrary to our predictions, parents who had experienced the challenging situation were not less, but more likely to use direct commands to elicit child compliance, compared to parents in the control condition. It might be that parents who previously experienced disruptive child behavior exercise more control over their children, granting them less autonomy than parents in a non-challenging situation (Verhoeven et al. 2010). As they might expect their children to be less compliant, these parents are focused on keeping control over their child’s behavior, even in the new situation. Yet, while parents in the challenging situation used more direct commands relative to control, they still used twice as many indirect commands as direct commands. This suggests that parents tend to formulate requests to their children more indirectly than directly.

### Limitations and Future Directions

This study has several limitations. First, the challenging situation was not only stressful for children, eliciting them to become more disruptive, but arguably also for parents themselves. Parents were not only connected to a physiological device which limited their abilities to follow the child, but they were also forced to terminate the pleasant playing situation and complete questionnaires while the child was not entertained. Thus, while our manipulation check showed that the situation indeed evoked disruptive behavior in children, we cannot rule out that changes in parent-child dynamics other than disruptive child behavior alone contributed to changes in parental self-efficacy, stress, and parenting behaviors. Because disruptive behaviors are rarely detached from challenging parenting situations, and parent-child interactions are always bidirectional, we chose to mimic an everyday challenging situation to enhance ecological validity. Second, while we aimed to measure how parental stress reactions impact subsequent parenting behaviors, our paradigm might have targeted feelings that were only linked to the momentary situation (i.e., the previous task). This rendered it difficult to disentangle feelings of stress during the task and lingering feelings of stress after the task. We chose this approach because our main question focused on parents’ immediate reactions to child disruptive behaviors. Future research might implement designs that allow to investigate the effects of lingering feelings of stress on parenting practices. Relatedly, how parents feel after a challenging situation might also reflect their ability to regulate stress responses. Initial findings indicate that well-regulated parents are more likely to use positive parenting strategies (Leerkes et al. 2016). Hence, future research might investigate individual differences in parents’ ability to regulate their feelings after a challenging situation, and how these regulation efforts in turn affect their parenting. Third, while our sample mainly included children with elevated levels of disruptive behavior, most parents were highly educated and lived together with a partner. Our results are therefore likely to only generalize to demographically advantaged families with disruptive toddler-age children. As an example, we do not know how well our findings generalize to families exposed to stressors that greatly influence daily-life functioning, such as unemployment, economic struggles, or being a single parent (Conger et al. 1992; Whitesell et al. 2015). These persistent stressors might influence parental state self-efficacy and stress in challenging parenting situations even more strongly. Similarly, parents with less disruptive children might react less strongly to challenging situations. Fourth, although we were well-equipped to detect separate moderation effects, our sample was relatively small to test the mediation model where both parental self-efficacy and stress reactivity underlie positive parenting in challenging situations. The relation between parental self-efficacy and positive affect approached significance, and should be further investigated in future studies with larger samples.

Despite these limitations, our study is among the first to adopt an experimental paradigm to shed light on how disruptive child behavior shapes parental state self-efficacy and stress. In addition, although thoughts of self-efficacy and feelings of stress are related, our approach to analyze them separately and simultaneously allowed us to demonstrate that both indeed play meaningfully different roles in how parents react to disruptive child behavior. Future research might investigate how parental states relate to more immediate parenting behaviors by examining dyadic interactions during challenging situations. For example, situational measures of parental stress levels, such as skin conductance responses (e.g., Wood et al. 2014), allow for investigating how phasic arousal underlies parents’ immediate behaviors to the child’s disruptive instance. Moreover, focused experimental studies (i.e., microtrials) can help to indicate how parental self-efficacy and stress impact other discrete parenting practices (e.g., providing reprimands or praise). Lastly, measuring parental state and trait behaviors across multiple time points might clarify the unique and interacting effects of states and traits in bidirectional parent-child interactions.

That parents display strong reactions to disruptive child behavior, particularly when they question their competence, might explain to some extent why parenting interventions tend to yield only small effects on disruptive child behavior (van Aar et al. 2017). If parents render it difficult to avoid negative thoughts and feelings in light of their child’s disruptive behavior, then teaching parenting skills alone may not be sufficient to change parental behavior in daily life. Our findings suggest that, even more than in current practice, parenting interventions need to enhance their focus on maintaining parents’ self-efficacious thoughts in challenging situations. There is emerging evidence that brief interventions can successfully boost parental thoughts of self-efficacy, and that heightened self-efficacy increases parental use of positive parenting strategies, in turn even reducing disruptive child behavior (Mouton and Roskam 2015).

## Conclusion

Disruptive child behavior has a strong negative impact on immediate parental self-efficacy and stress responses, both perceived and physiologically. This impact is most adverse for parents who seem most vulnerable, such as parents who experience low levels of self-efficacy in daily life. Addressing parental thoughts of self-efficacy in response to challenging situations may help parents to maintain positive parent-child interactions, potentially reducing the risk of negative cycles of interaction and higher rates of disruptive behavior among young children.

## Electronic supplementary material

ESM 1 (DOCX 15 kb)

ESM 2 (DOCX 18.1 kb)

ESM 3 (DOCX 19 kb)
