# Supplementary material for: Parental Reactivity to Disruptive Behavior in Toddlerhood: An Experimental Study
Source: J Abnorm Child Psychol. 2018 Oct 29;47(5):779–90. doi: 10.1007/s10802-018-0489-4 (PMC6469638; doi:10.1007/s10802-018-0489-4)
Supplement: Supplementary file 1 — (DOCX 15 kb) [file 10802_2018_489_MOESM1_ESM.docx]

Parental Reactivity to Disruptive Behavior in Toddlerhood: An Experimental Study, *Journal of Abnormal Child Psychology*

**Online Resource 1**

**Questionnaire on parental levels of state self-efficacy and distress during Task 2**

**In the last task, you filled in questionnaires while your child was in the room with you.**

***How did you feel during this task?***

1 = not at all 5 = to a large extent

| Stressed | 1 2 3 4 5 |
| --- | --- |
| Tensed | 1 2 3 4 5 |
| Frustrated | 1 2 3 4 5 |
| Nervous | 1 2 3 4 5 |
| Relaxed | 1 2 3 4 5 |
| Comfortable | 1 2 3 4 5 |
| At ease | 1 2 3 4 5 |

***How well do you feel that you managed the previous task as a parent?***

1 = not at all 5 = to a large extent

| I managed it well. | 1 2 3 4 5 |
| --- | --- |
| This task was easy for me. | 1 2 3 4 5 |
| I did a good job. | 1 2 3 4 5 |
| This task was difficult for me. | 1 2 3 4 5 |
| I managed it badly. | 1 2 3 4 5 |
| I did a poor job. | 1 2 3 4 5 |

**Further instructions:**

**We will quickly start with the next task. For that, please make sure that your child clears up all the toys, he/she is playing with at the moment. Your child can put them in the box next to it. I’m afraid we cannot let you help your child clear up.**
