# Supplementary material for: Parental Reactivity to Disruptive Behavior in Toddlerhood: An Experimental Study
Source: J Abnorm Child Psychol. 2018 Oct 29;47(5):779–90. doi: 10.1007/s10802-018-0489-4 (PMC6469638; doi:10.1007/s10802-018-0489-4)
Supplement: Supplementary file 2 — (DOCX 18.1 kb) [file 10802_2018_489_MOESM2_ESM.docx]

Parental Reactivity to Disruptive Behavior in Toddlerhood: An Experimental Study, *Journal of Abnormal Child Psychology*

**Online Resource 2**
*Correlations* *Between Study Variables for Experimental and Control Group.*

| Variable | 1 | | 2 | | 3 | | 4 | | 5 | | 6 | | 7 | | 8 | |
| --- | --- | --- | --- | --- | --- | --- | --- | --- | --- | --- | --- | --- | --- | --- | --- | --- |
| 1 State self-efficacy |  |  | −.47 | *** | −.01 |  | .53 | *** | −.20 |  | −.15 |  | .01 |  | .11 |  |
| 2 State distress | −.37 | ** |  |  | .09 |  | −.26 |  | .12 |  | .14 |  | −.00 |  | −.01 |  |
| 3 Arousal | .08 |  | −.12 |  |  |  | .00 |  | −.20 |  | −.30 | * | −.17 |  | .08 |  |
| 4 Trait self-efficacy | .21 |  | −.04 |  | .24 |  |  |  | −.18 |  | −.16 |  | −.10 |  | .09 |  |
| 5 Trait distress | −.23 |  | .55 | *** | −.30 | * | −.46 | *** |  |  | .33 | * | .38 | * | .33 | * |
| 6 Baseline arousal | .09 |  | .17 |  | −.02 |  | .10 |  | .12 |  |  |  | −.08 |  | .08 |  |
| 7 Direct command | .41 |  | −.31 |  | −.16 |  | .06 |  | −.31 |  | .08 |  |  |  | .33 |  |
| 8 Positive affect | .02 |  | −.06 |  | −.09 |  | .02 |  | .06 |  | .18 |  | .33 |  |  |  |

*Note.* Correlations above the diagonal represent results for the experimental group (*n* = 56); correlations below the diagonal represent results for the control group (*n* = 54).

*** *p* < .001 ** *p* < .01 * *p* < .05
