# Supplementary material for: Parental Reactivity to Disruptive Behavior in Toddlerhood: An Experimental Study
Source: J Abnorm Child Psychol. 2018 Oct 29;47(5):779–90. doi: 10.1007/s10802-018-0489-4 (PMC6469638; doi:10.1007/s10802-018-0489-4)
Supplement: Supplementary file 3 — (DOCX 19 kb) [file 10802_2018_489_MOESM3_ESM.docx]

Parental Reactivity to Disruptive Behavior in Toddlerhood: An Experimental Study, *Journal of Abnormal Child Psychology*

**Online Resource 3**
*Comparison of all SEM Models.*

| Model | χ^2^ | *p* | RMSEA | CFI |
| --- | --- | --- | --- | --- |
| Baseline mediation model | 35.142 | <.001 | .219 | .501 |
| Mediation model including covariances | 8.108 | .088 | .101 | .930 |
| Partial mediation model (final model) | 1.619 | .655 | <.001 | >.999 |
